# Effects of Neuromuscular Electrical Stimulation Combined with Exercises versus an Exercise Program on the Pain and the Function in Patients with Knee Osteoarthritis: A Randomized Controlled Trial

**DOI:** 10.1155/2013/272018

**Published:** 2013-09-14

**Authors:** Aline Mizusaki Imoto, Stella Peccin, Kelson Nonato Gomes da Silva, Lucas Emmanuel Pedro de Paiva Teixeira, Marcelo Ismael Abrahão, Virgínia Fernandes Moça Trevisani

**Affiliations:** ^1^Internal Medicine Department, Federal University of São Paulo, 04023-900 São Paulo, SP, Brazil; ^2^Human Movement Science Department, Federal University of São Paulo, 11060-001 Santos, SP, Brazil; ^3^Medicine Department, Santo Amaro University, 04829-300 São Paulo, SP, Brazil

## Abstract

*Objectives*. To investigate the effect of 8 weeks of NMES + Ex (neuromuscular electrical stimulation combined with exercises) on pain and functional improvement in patients with knee osteoarthritis (OA) compared to exercise (Ex) alone. *Design*. Randomized controlled trial. *Setting*. A specialty outpatient clinic. *Participants*. Patients (*N* = 100; women = 86, men = 14; age range, 50–75 years) with knee OA. *Interventions*. Participants were randomly assigned to NMES + Ex or Ex group. *Outcome Measures*. Numerical Rating Scale 0 to 10 (NRS) and the Timed Up and Go (TUG) test were the primary outcomes. The secondary outcomes used were the Western Ontario and McMaster Universities Osteoarthritis Index (WOMAC). *Results*. Following the interventions, a statistically significant improvement in both groups was observed in all outcomes assessed. For the comparison between the groups, no statistically significant difference was found between the NMES + Ex and the Ex groups in NRS (*P* = 0.52), TUG test (*P* = 0.12), and aspects of WOMAC: pain (*P* = 0.26), function (*P* = 0.23), and stiffness (*P* = 0.63). *Conclusion*. The addition of NMES to exercise did not improve the outcomes assessed in knee OA patients. This study was registered at the Australian Clinical Trials Registry (ACTRN012607000357459).

## 1. Introduction

Osteoarthritis (OA) is the most common form of arthritis. It affects one-third of adults and tends to increase with age [1]. Knee OA is associated with symptoms of pain, swelling, instability, and reduced range of motion (ROM). These symptoms lead to functional impairment, increasing the risk of morbidity and mortality [2, 3].

The synovium is infiltrated with inflammatory cells and secretes excess synovial fluid, leading to capsular swelling [4]. Through a spinal reflex, the capsular swelling inhibits muscle activation, which, combined with disuse, may cause muscle weakness and atrophy [1]. Because the quadriceps muscle acts as shock absorber for the knee joint, weakness in the thigh muscle reduces joint protection, resulting in overload [4]. Exercises strengthen the muscles, reduce pain, improve physical function, and are therefore considered a major intervention in the conservative treatment of patients with knee OA [5]. In addition to muscle strengthening exercises, stretching exercises are commonly used to increase ROM and are often prescribed in rehabilitation protocols as part of routine warm-up to prepare the muscles and joints for other types of exercise, such as aerobic and strengthening programs [6, 7]. Stretching of the hamstring muscles may improve knee extension ROM in OA patients. 

Neuromuscular electrical stimulation (NMES) is defined as the application of electrical stimulation using surface electrodes placed over skeletal muscles to produce visible muscle contraction through the activation of intramuscular nerve branches [8]. This technique can also be used as a form of physical therapy in the treatment of patients with knee OA. The goals of rehabilitation protocols that include NMES are to provide additional stimulus to increase muscle strength in patients with knee OA [9]. The methods and findings of previous studies on the effectiveness of NMES in knee OA differ in the modulation of NMES parameters, choice of the outcomes used to evaluate the patients, and characteristics of the control groups. This leads to a lack of consensus regarding the effectiveness attained from including NMES in conventional rehabilitation protocols. Major flaws with regard to methodological quality were found in clinical trials testing the use of NMES in the conservative treatment of patients with knee OA. Only one of these studies reported using allocation concealment, blinded assessment, and intention-to-treat analysis [10]. 

Thus, the objective of this study was to conduct a randomized clinical trial following methodological criteria, including allocation concealment, blinding of the examiner, and application of intention-to-treat analysis to assess the role of NMES in improving pain and physical function in patients with knee OA. It is important to study interventions with the potential to improve functional status in this patient population [11]. 

## 2. Methods

The study was conducted at the Interlagos Specialty Outpatient Clinic, São Paulo, Brazil. Patients were referred from the Rheumatology Department according to the inclusion and exclusion criteria and randomly allocated into groups using a computer-generated randomization chart. The allocation codes were sealed in opaque envelopes by a third person not involved in the study to avoid selection bias. 

Written informed consent was obtained from all participants. The study was approved by the Research Ethics Committee of the Universidade Federal de São Paulo (UNIFESP), Brazil, no. 0141/07, and registered with the Australian Clinical Trials Registry, no. ACTRN012607000357459.

Setting the significance level at 5% and the power of the sample at 80%, a sample size of 40 patients per group was estimated to be necessary to detect a difference of at least 1 minute ± 3 seconds in the Timed Up and Go (TUG) test, which was considered to be the minimum clinically significant difference for the present trial [12]. Paired Student's *t*-test and analysis of covariance (ANCOVA) were used for comparisons between groups; the covariant was obtained from a previous study [13].

One hundred patients were recruited according to inclusion and exclusion criteria. Eligibility criteria were age 50 to 75 years, OA grade 2 or greater according to the radiographic classification of OA proposed by Kellgren and Lawrence [14], and diagnosis of knee OA based on the American College of Rheumatology (ACR) criteria. Exclusion criteria were use of a pacemaker, unstable heart conditions, participation in another physical activity program, inability to exercise on a stationary bicycle ergometer, inability to walk, previous hip or knee arthroplasty, diagnosis of fibromyalgia, epilepsy, and skin tumor or lesion at the NMES application site. 

Patients were divided into two groups of 50 each: (1) the NMES combined with exercises (NMES + Ex) group and (2) exercise (Ex) group. Patient medication was standardized and not modified during the study period. Paracetamol was prescribed for pain, and diacerein and chloroquine for OA control.

Interventions were delivered for both groups by the same physical therapist, twice a week, for 8 weeks, with each session lasting about 40 minutes. 

All patients received a manual including guidelines on how not to overload the knee during daily activities and instructions on the use of ice packs in case of pain and inflammation and warm compresses in case of pain without inflammation as follows. 

*Manual for Patients with Osteoarthritis of the Knee.* The purpose of this manual is to explain osteoarthritis and to teach how you can adjust yourself to your daily activities, according to the knee symptoms. 

Try to seriously follow our orientations for your own benefit!

*The Knee.* The knee joint is composed of 3 bones—the femur (thigh bone), the patella (kneecap) and the tibia (leg bone). It has muscles, capsule, ligaments, meniscus, and the cartilage that lines the bones and protects them from the impact. The knee joint supports nearly the whole weight of our body. 

*What Is Osteoarthritis?* It is a disease caused by the breakdown of cartilage in the joints. The layers in the cartilage become damaged and with time they lose the function of smoothing the contact between the bone surface and the joints. The pain is a result of the attrition of one bone against the other in the absence or decreased cartilage in the joints.

*What Are the Signs and Symptoms?* Patients with osteoarthritis may have some pain mainly when starting a movement, as in morning stiffness or after immobilization. With time, the pain might be intensified and be permanent. The presence of crepitation when moving the knees is often.

*What Kind of Difficulties Might I Have in My Daily Life?* Difficulties found in daily live vary according to the patients' symptoms. In general, however, the patient has pain and difficulty when supporting the body weight using the affected knee, going up and down the stairs, or when walking.

*What Should I Do When It Is Painful?* A doctor can prescribe the treatment for osteoarthritis. However, a simple form of improving the pain is to use warm to hot water bottle over the knee joint (be careful not to burn the skin, use a protection, and test the water temperature before using it.

*What If It Is Swollen?* To manage the swollen, you can combine rest, use of ice pack, and elevating the leg above the level of the heart. The ice pack should be placed over the knee joint for 20 minutes.

*What Are the Other Recommendations?*

- If you are overweight, losing some kilos will reduce the stress over the joint.
- Wear comfortable shoes with a rubber sole and no heels.
- In case of pain when walking, use a cane as an aid tool.
- Try to have a good night sleep.

### 2.1. NMES Combined with Exercise Group

Treatment for patients in the NMES + Ex included 10 minutes on a stationary bicycle, stretching of hamstring muscles (3 repetitions of 30 seconds) with the aid of an elastic band, and loaded quadriceps strengthening exercises combined with NMES. The strengthening exercise with NMES was performed in the sitting position with the knee and hip flexed to 90 degrees; patients contracted their quadriceps at each NMES stimulus. 

NMES was applied using an electrical stimulator (Globus ACTIVA 600 Pro, Globus, Italia) with two 7.5 × 13 cm self-adhesive electrodes (ValuTrode electrodes, Axelgaard Manufacturing Co. Ltd., Fallbrook, CA) placed over the region of the quadriceps muscle (rectus femoris and vastus medialis). NMES parameters were as follows: pulsed current, biphasic, asymmetrical, rectangular waveform, frequency 50 Hz, pulse duration 250 *μ*s, contraction time 10 s, rest time 30 s every 20 minutes; current intensity was the maximum tolerated by each patient [15]. 

### 2.2. Exercise Group (Ex)

Patients in the Ex group performed the same exercise program as those in the NMES group but without NMES. The exercise protocol included 10 minutes of warm-up on a stationary bicycle ergometer, stretching of hamstrings muscles with the aid of an elastic band, and knee extension exercises performed for 3 sets of 15 repetitions with rest intervals of 30–45 seconds between sets. 

For both groups, the training load for the strengthening exercises was established based on 50–60% of the 10-repetition maximum (RM) instead of 1 RM to avoid injury by excessive muscle contraction [16].

### 2.3. Outcomes

Patients were evaluated before and after intervention by a physical therapist blinded to group assignment. The primary outcomes were the TUG test results [12] and pain walking on a flat surface in the last 72 h measured on an 11-point Numerical Rating Scale (NRS) [17]. The secondary outcomes were scores on the pain, physical function, and stiffness subscales of the culturally validated Brazilian-Portuguese version of the Western Ontario and McMaster Universities Osteoarthritis Index (WOMAC) [18, 19]. In this study, WOMAC pain, physical function, and stiffness scores were analyzed separately. 

### 2.4. Statistical Analysis

Paired Student's *t*-test was performed to compare pre- and postintervention values at a significance level of 0.05 (*P* < 0.05). Statistical analysis was performed on an intent-to-treat (ITT) basis and included all patients who were randomized to treatment. Mixed model analysis of variance with repeated measures was used with occasion measures as within-group factors and intervention as between-group factor. 

Relations between the observations were analyzed using an unstructured covariance matrix. Missing-data imputation was not performed to evaluate pre- and postintervention differences between the two groups, because Chakraborty and GU [20] showed that mixed model analysis without missing-data imputation always provides equal or more power than does mixed model analysis with missing-data imputation. Effect size was calculated as the difference between the means divided by the standard deviation using Cohen's *d* [21]. The analyses were performed using the General Linear Model (GLM), and mixed analyses were carried out using the Statistical Analysis Software (SAS) version 9.2 for Windows [22].

## 3. Results

**Figure 1:**
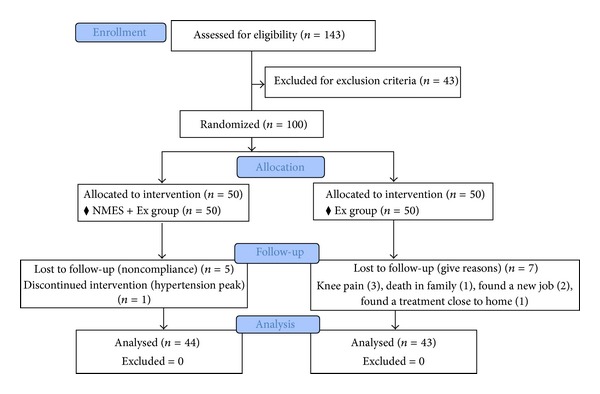
Flowchart showing the number of patients randomized and evaluated in each group.

The demographic and clinical characteristics of patients, including age, sex, side treated, and body mass index (BMI), as well as TUG test values, NRS pain scores, and WOMAC scores on the pain, physical function, and stiffness subscales, are shown in Table 1. Eighty-seven patients completed the study. Of the 13 dropouts, 6 (12%) patients were in the NMES + Ex group and 7 (14%) in the Ex group (Figure 1). 

### 3.1. Primary Outcomes

No significant differences between groups were found in NRS pain scores and TUG test time on ITT analysis. A significant decrease in pain intensity (NRS scores) and TUG test time was observed after intervention compared with baseline in the NMES + Ex group (NRS scores, *P* < 0.0001; TUG test, *P* < 0.0001) and Ex group (NRS scores, *P* < 0.0001; TUG test, *P* < 0.0001). The primary outcomes are listed in Table 2. 

### 3.2. Secondary Outcomes

No significant differences between groups were found on the pain, physical function, and stiffness subscales of the WOMAC index on ITT analysis. There was a significant improvement in all WOMAC subscales in the NMES + Ex group (pain; *P* < 0.0001; physical function, *P* < 0.0001; stiffness, *P* < 0.0001) and in the Ex group (pain, *P* < 0.0001; physical function, *P* < 0.0001; stiffness, *P* = 0.0009). The secondary outcomes are listed in Table 2.

### 3.3. Adverse Effect

One patient in the NMES + Ex group exhibited a blood pressure spike, which may have resulted from the use of NMES or from the exercise program itself. The following contraindications to the use of NMES were respected: avoiding the use of NMES over areas of tumor, with open wounds, or bleeding, and in patients with pacemakers [15].

## 4. Discussion

In this randomized clinical trial, the NMES combined with exercise did improve pain and physical function, but there was no evidence that it did better than exercise alone. Our results are in agreement with the findings of Rosemffet et al. [23] who conducted a pilot study comparing the treatment results of patients with knee OA (*n* = 26) treated with either NMES alone or exercises alone or exercises combined with NMES. The authors reported a significant improvement on the WOMAC pain subscale in the three groups, but no significant differences in WOMAC scores were found between groups [23]. 

The lack of difference in treatment outcome between groups in the present study might be attributed to the fact that the participants had no clinically significant muscle or functional impairment. A finding substantiating this hypothesis is that the mean TUG test values in the two groups were similar to those found in the study of Steffen et al., for elderly patients with no physical limitations categorized under the same mean age group [24]. The TUG test values reported were 8 ± 2 seconds for patients with 60–69 years old and 9 ± 3 seconds for patients with 70–79 years old. In the present study, the baseline values for TUG test were 8.27 + 1.76 for NMES + Ex group and 9.34 ± 2.47 for Ex group.

Given that the greater the muscle impairment, the greater the NMES effect, patients with a more advanced stage of OA might obtain greater benefit from NMES [25–27]. With regard to evidence on the effectiveness of NMES in the rehabilitation of patients with knee OA, the Cochrane Collaboration published a systematic review evaluating the pre- and postoperative use of NMES in total knee arthroplasty [28]. The two studies that were evaluated were classified as being at high risk of bias, because randomization and allocation concealment were not described, and no data obtained from the study groups were presented in terms of means and standard deviations. It was not possible to determine whether or not the pre- and postoperative use of NMES was effective in the rehabilitation of knee arthroplasty patients, and therefore further studies are warranted. Regarding lines of evidence on the effectiveness of NMES in the treatment of other conditions, Kim et al. [29] conducted a review of the use of NMES after anterior cruciate ligament (ACL) reconstruction. Based on eight different studies, the authors concluded that the use of NMES combined with exercises can be effective in improving quadriceps strength up to four weeks after surgery. However, there was no sufficient evidence to affirm that NMES has any positive effect on the functional performance of patients who had undergone ACL reconstruction [29]. No literature review could be found on the effectiveness of NMES in patients with knee OA without indication for knee arthroplasty.

A limitation of this study is that the current intensity used for electrical stimulation was not recorded. However, the maximum current intensity tolerated by each patient was applied as in previous studies [9, 24, 30]. No participant showed intolerance to electrical stimulation. 

The questionnaires used in this study have been translated into Brazilian Portuguese, cross-culturally adapted, and validated in previous studies [19, 31, 32]. The assessment of patient-reported pain intensity and physical function was performed according to the international consensus on outcomes measures for phase III clinical trials in OA, in which pain and physical function are identified as the most important outcome measures in randomized clinical trials [18]. The TUG test was chosen because it is a simple and inexpensive test designed to assess the functional mobility of patients based on activities of daily living [12].

Statistical analysis was performed on an intent-to-treat basis to minimize the impact of protocol violations (which may occur after randomization) on the results and conclusions and to avoid an overestimation of the treatment effect. This randomized clinical trial conforms to the Consort Statement (Consolidated Standards of Reporting Trials) [33], whose aim is to improve the quality of reports of randomized clinical trials. Given the predominance of mild and moderate OA cases among the participants, the results of this study can be generalized especially for patients with similar severity levels of OA.

## 5. Conclusions

Our results revealed that the application of NMES combined with a conventional exercise program was as effective as the exercise program alone in reducing pain and improving physical function in patients with knee OA, and therefore no therapeutic benefit was observed with the use of NMES. 

## 6. Clinical Implications

Moderate exercises, including warm-up and muscle stretching and strengthening exercises—combined or not with NMES—are recommended to reduce pain and improve physical function and quality of life in patients with knee OA.

## Figures and Tables

**Table 1:** Baseline characteristics of subjects.

Characteristics	NMES + Ex	Ex
*N*	50	50
Age*	60.60 ± 6.72	61.50 ± 6.94
Female^†^	46	40
Male^†^	4	10
Treated leg^†^		
Right	40	40
Left	34	34
Both sides	26	26
BMI*	30.08 ± 3.80	29.72 ± 4.11
KL grade^†^		
2	95.35	92.68
3	2.33	4.88
4	2.33	2.44
NRS (0–10)*	7.06 ± 1.95	7.42 ± 2.01
TUG Test (seconds)*	8.27 ± 1.76	9.34 ± 2.47
WOMAC pain score*	8.72 ± 4.20	10.32 ± 3.54
WOMAC stiffness score*	3.64 ± 2.15	3.66 ± 2.64
WOMAC function score*	28.54 ± 13.96	35.15 ± 11.88

Note. *Data are presented as mean ± SD. ^†^Data are presented as %. Abbreviations: BMI: body mass index, KL: Kellgren and Lawrence; NRS: Numerical Rating Scale; TUG: Timed Up and Go; WOMAC: Western Ontario and Mcmaster Universities. NMES + Ex: NMES combined with exercise, Ex: Exercise, NRS: Numerical Rating Scale; TUG: Timed Up and Go Test, WOMAC: Western Ontario McMaster Universities Osteoarthritis Index.

**Table 2:** Changes within and between groups (ITT analysis).

Outcomes	NMES + Ex		Ex		Difference between means (95% CI), effect size	*P* value between groups
	At 8 weeks	Change (95% CI)	At 8 weeks	Change (95% CI)		
NRS (0–10)*	4.30 ± 3.01	−2.70 (−3.56 to −1.84)^†^	4.27 ± 2.45	−3.17 (−4.23 to −2.10)^†^	0.42 (−0.87 to 1.72), 0.15	0.52
TUG*	6.77 ± 1.08	−1.36 (−1.84 to −0.87)^†^	7.42 ± 1.70	−2.00 (−2.54 to −1.46)^†^	0.56 (−0.15 to 1.27), 0.39	0.12
WOMAC pain*	5.64 ± 4.33	−2.97 (−4.22 to −1.72)^†^	6.29 ± 3.96	−3.87 (−5.02 to −2.72)^†^	0.92 (−0.71 to 2.55), 0.23	0.26
WOMAC stiffness*	2.39 ± 2.24	−1.34 (−1.9 to −0.74)^†^	2.10 ± 2.26	−1.51 (−2.36 to −0.65)^‡^	0.25 (−0.78 to 1.29), 0.10	0.63
WOMAC function*	20.91 ± 14.06	−8.02 (−11.34 to −4.69)^†^	23.83 ± 15.49	−10.95 (−14.84 to −7.05)^†^	3.14 (−2.02 to 8.29), 0.26	0.23

Abbreviation: *data are presented as mean ± SD. ITT: intention to treat; CI: confidence interval; NMES + Ex: NMES combined with exercise, Ex: exercise; NRS: Numerical Rating Scale; TUG: Timed Up and Go Test; WOMAC: Western Ontario McMaster Universities; ^†^ *P* < 0.05 denotes a statistical significant difference, ^‡^ *P* < 0.0001.

## List of Abbreviations

ACTRN: Australian Clinical Trials Registry Number
ACR: American College of Rheumatology
ANCOVA: Analysis of covariance
BMI: Body mass index
CONSORT: Consolidated standards of reporting trials
Ex: Exercises
GLM: General linear models
ITT: Intention-to-treat analysis
NMES + Ex: Neuromuscular electrical stimulation combined with exercises
NRS: Numerical Rating Scale
NMES: Neuromuscular electrical stimulation
OA: Osteoarthritis
RM: Repetition maximum
ROM: Range of motion
SAS: Statistical analysis software
SD: Standard deviation
TUG: Timed Up and Go
UNIFESP: Universidade Federal de Sao Paulo
WOMAC: Western Ontario and McMaster Universities Osteoarthritis Index.
